# Functional lung imaging in thoracic tumor radiotherapy: Application and progress

**DOI:** 10.3389/fonc.2022.908345

**Published:** 2022-09-23

**Authors:** Pi-Xiao Zhou, Shu-Xu Zhang

**Affiliations:** ^1^ Radiotherapy Center, Affiliated Cancer Hospital & Institute of Guangzhou Medical University, Guangzhou, China; ^2^ Department of Oncology, The First People's Hospital of Changde City, Changde, China

**Keywords:** functional lung, ventilation (V), perfusion (Q), radiotherapy, four-dimensional CT, magnetic resonance imaging (MRI), single-photon emission computed tomography (SPECT), positron emission tomography (PET)

## Abstract

Radiotherapy plays an irreplaceable and unique role in treating thoracic tumors, but the occurrence of radiation-induced lung injury has limited the increase in tumor target doses and has influenced patients’ quality of life. However, the introduction of functional lung imaging has been incorporating functional lungs into radiotherapy planning. The design of the functional lung protection plan, while meeting the target dose requirements and dose limitations of the organs at risk (OARs), minimizes the radiation dose to the functional lung, thus reducing the occurrence of radiation-induced lung injury. In this manuscript, we mainly reviewed the lung ventilation or/and perfusion functional imaging modalities, application, and progress, as well as the results based on the functional lung protection planning in thoracic tumors. In addition, we also discussed the problems that should be explored and further studied in the practical application based on functional lung radiotherapy planning.

## Introduction

Radiotherapy occupies an essential role in tumor treatment. Approximately 50% of cancer patients require radiotherapy at a certain stage of the entire disease course, and it accounts for 40% of the tumor cure rate (1). In the past, radiotherapy was based on the assumption of the consistency of spatial tissue function without considering the heterogeneity of tissue function (2). However, due to the organ’s structure or the disease, functional heterogeneity is likely to exist (such as in the lung, liver, and parotid gland) (3, 4). Previous studies have demonstrated that lung function distribution in diverse lung tissue regions of patients with lung cancer is uneven (3, 5). Different locations and functional states respond differently to radiation (i.e., the better the functional state, the more sensitive) (3, 6). The degree of regional lung function decline after radiotherapy has a positive linear relationship with the radiation dose in this area (7). In the radiotherapy of thoracic tumors (such as lung cancer, and esophageal cancer), the existence of radiation-induced lung injury (RILI) not only limits the increase in tumor target dose but also seriously affects patients’ quality of life after radiotherapy (8). Furthermore, the risk of RILI is further increased in patients with older age, smoking, chronic obstructive pulmonary disease, interstitial lung disease, and concurrent chemotherapy (9).

In recent decades, more clinical evidence has shown the crucial role of functional imaging in tumor target delineation (10). Furthermore, the introduction of multi-detector computed tomography (MDCT), four‐dimensional CT (4DCT), dual-energy CT (DECT), magnetic resonance imaging (MRI), single-photon emission CT (SPECT), and positron emission tomography (PET) functional imaging modalities is of significant benefit in the design and evaluation of radiotherapy planning. Studies have shown that integrating functional imaging into radiation treatment planning can reduce the incidence of RILI by limiting the dose of functional lung irradiation (3, 11). This manuscript reviewed the principle, progress, and clinical application of multiple functional imaging modalities used to detect lung ventilation or perfusion function. It also discusses the problems that should be considered in practical applications and should be further explored and resolved.

## Lung ventilation and perfusion

Air and blood are the fundamental participants in the gas exchange process, and the two must be maintained in an appropriate ratio to ensure sufficient and adequate progress (2). Pulmonary ventilation function measurement is the amount of air inhaled or exhaled by the lungs per time. In clinical, pulmonary function tests (PFT) are commonly used to evaluate patients’ ventilatory function, which usually reflects that the overall function of the lung cannot distinguish the differences between local lung functions and the sensitivity to early functional changes of the disease is limited (12). Numerous invasive techniques or radioisotope imaging have been tried to directly or indirectly quantify regional ventilation. However, these methods are restricted in clinical use by invasiveness and poor temporal and spatial resolution (13). Lung ventilation functional imaging was primarily used to detect pulmonary embolism, asthma, and chronic obstructive pulmonary disease (14, 15). It was relatively late when combined with radiotherapy to protect the lung function.

Pulmonary perfusion function measurement refers to the blood flow of lung tissue per time. Ventilation and perfusion can influence each other through a series of mechanisms, such as lack of adequate ventilation to cause hypoxia contraction of blood vessels, which in turn causes a decrease in matched perfusion (2, 4). Other diseases (pulmonary embolism, tumor-causing bronchial or aortic obstruction) can cause mismatch defects (2, 16). Lung ventilation/perfusion functional imaging is a two- or three-dimensional map showing the changes in lung volume during the entire respiratory cycle/blood flow distribution (2). The corresponding color-coded maps can be generated to observe the differences in overall lung function distribution more intuitively. The excessive radiation exposure of lung tissue leads to RILI, which is physiologically characterized by a reduction of airflow (ventilation) and blood flow (perfusion) (17).

## Functional lung imaging modalities

### Computed tomography

#### Multidetector CT

The previous CT imaging speed was slow, and it was difficult to use in clinical practice. However, electron beam CT (EBCT) uses parallel X-ray targets to obtain higher scanning speeds, a valuable tool for ventilation imaging and perfusion imaging, but it is expensive (18, 19). Until the advent of MDCT, scanning speed and imaging resolution have been greatly improved, so a noninvasive lung ventilation function method that is determined based on the wash-in and/or wash-out rate of the nonradioactive gas xenon (Xe) and forms a color-coded image of regional ventilation has been developed (13, 19–21). It means that the CT intensity of the lung image can be used to calculate the local air volume distribution at the two lung volumes, and through image registration, the mapping between the two images is obtained to get the regional ventilation change of the object (22). There are some problems in practical applications: one is radiation dose; another is the need to track the gas washing in and/or washing out at a fixed chest level, which limits the coverage of the lungs on the *z*-axis; the third is the image registration error; and the fourth is the accuracy of ventilation measurement in restricted areas where Xe will dissolve in the blood (15, 19, 23, 24). Moreover, Mahnken et al. (24) showed in a rabbit study that although krypton has a worse enhancement effect than Xe ventilation and requires a higher concentration, it is feasible to evaluate lung ventilation in MDCT.

In addition, Eslick et al. (25) directly performed an expiratory/inspiratory breath-hold CT scan on the patient. They proved that the lung ventilation function image obtained based on the change of Hounsfield unit (HU) has good consistency with the ventilation image obtained by PET. Moreover, MDCT and iodine-containing contrast agents can be used to assess regional lung perfusion dynamically, but the limited scanning range is its main drawback (13, 26).

#### Dual-energy CT

According to the imaging principle of DECT, it can be divided into two categories: one is through an X-ray tube to achieve energy separation (rapid KV switch CT, dual-source CT, twin-beam CT), and the other is achieved by the detector (dual-layer detector CT) (26–29). DECT can obtain two datasets of different energies at the same time through one acquisition, and a series of postprocessing techniques reconstruct them into virtual monoenergetic imaging (removing metal artifacts and improving image quality) and basic material imaging (substance separation and quantification) (30–32). In the past, DECT was mainly used for diagnosis and differential diagnosis, characterized tumor differentiation and gene expression, staging, and evaluating prognosis (33).

Through the introduction of DECT, it is possible to inhale a mixture of Xe and oxygen in one breath and hold the breath for imaging, and then use the basic material decomposition algorithm to separate the Xe signal from other signals for lung ventilation imaging reconstruction (15, 34). Fuld et al. (23) confirmed that proper calibration and the use of 40%Xe/40%He/20%O_2_ mixed gas could reduce the influence of gravity on the distribution of Xe and help with accurate quantitative measurement. However, Xe ventilation imaging was limited to high radiation doses and adverse effects of Xe, and it requires special equipment to transfer (14, 35). Chung et al. (36) then studied the effects of a series of krypton concentrations to enhance ventilation and confirmed that it is feasible to replace Xe to perform DECT lung ventilation imaging in rabbits when the krypton concentration is greater than 70%, but there are also some drawbacks. Furthermore, Zhang et al. (35) evaluated the feasibility of using DECT to evaluate lung ventilation after aerosol inhalation of iodine-containing contrast agents in rabbits. The results showed that the findings on the DECT ventilation image were highly consistent with the changes in lung parenchyma on the thin-section CT image and histopathological findings. Iodine-enhanced DECT ventilation imaging has the advantages of high spatial resolution and easier implementation, but the safety of inhalation of iodinated contrast agents in humans has not been verified.

The DECT lung perfusion image can be performed after injecting an iodine-containing contrast agent. The iodine map can be formed according to the basic material decomposition algorithm principle to evaluate the blood perfusion (35). Studies have proved that the pulmonary perfusion blood volume assessed by DECT is reliable and can replace the actual regional lung parenchymal perfusion measurement (15, 37). Through the optimization of the contrast media injection parameters (including the use of saline tracker bolus), high concentration (>300 mg I/ml), holding on breath, and performing DECT scans in the direction of the caudal cranium, the lung perfusion image quality can be improved (26, 38). Si-Mohamed et al. (39) quantitatively evaluated the relative lobe perfusion correlation between the DECT iodine map and SPECT/CT perfusion image. The Pearson correlation coefficient was 0.93 (*r* = 0.93), which indicated a high similarity in the shape and severity of perfusion defects. In another study, Bahig et al. (40) also confirmed that the Pearson correlation coefficient is 0.89 (*r* = 0.89), and the average of fV_5_ (volume of functional lung receiving ≥5 Gy) and mean functional lung dose (f-MLD) between the anatomical and functional lung volumes difference is statistically significant, which shows that it is necessary to optimize the radiotherapy planning to protect the functional lung. Although a clear iodine threshold has not been established to delimit functional and nonfunctional lungs, this paves the way for the next step in designing DECT-based functional radiotherapy planning.

As mentioned above, the actual physiology of lung function is gas exchange (involving proper ventilation and perfusion ratio). However, in our opinion, there are probably no functional imaging methods that can be used to detect ventilation and perfusion functions simultaneously because they require inhalation of a gas tracer or intravenous contrast agent, and it will increase the radiation dose exposure and scanning time. However, the emergence of DECT realized this process, Hong et al. (41) proved that, based on the DECT basic material decomposition algorithm, the krypton and iodine concentrations can be obtained at the same time through one scan to achieve the assessment of lung ventilation and perfusion function.

#### Four‐dimensional CT

The 4DCT scan consists of a series of CT image sets taken at particular points throughout the entire respiratory cycle (usually 10 respiratory phases), and the data used for lung ventilation function imaging reconstruction are usually obtained through the end-inspiratory and end-expiratory phases (2, 42). The real-time position management system (RPM) is commonly used to monitor the patient’s respiratory movements (based on an infrared camera). 4DCT lung function ventilation images are obtained by CT images of different respiratory stages for deformation image registration (DIR), and then the ventilation imaging algorithm (VIA) is used to quantitatively calculate the changes (3, 43, 44). In order to guarantee the accuracy of 4DCT lung ventilation measurement, studies have compared with clinical PFT, MRI, SPECT, and PET lung ventilation imaging, all suggesting that 4DCT ventilation can provide reliable lung function assessment (45–49). In addition, 4DCT can also remove motion artifacts and reflect the tumor’s internal target volume (ITV) for individualized radiotherapy planning (50).

**Figure 1:**
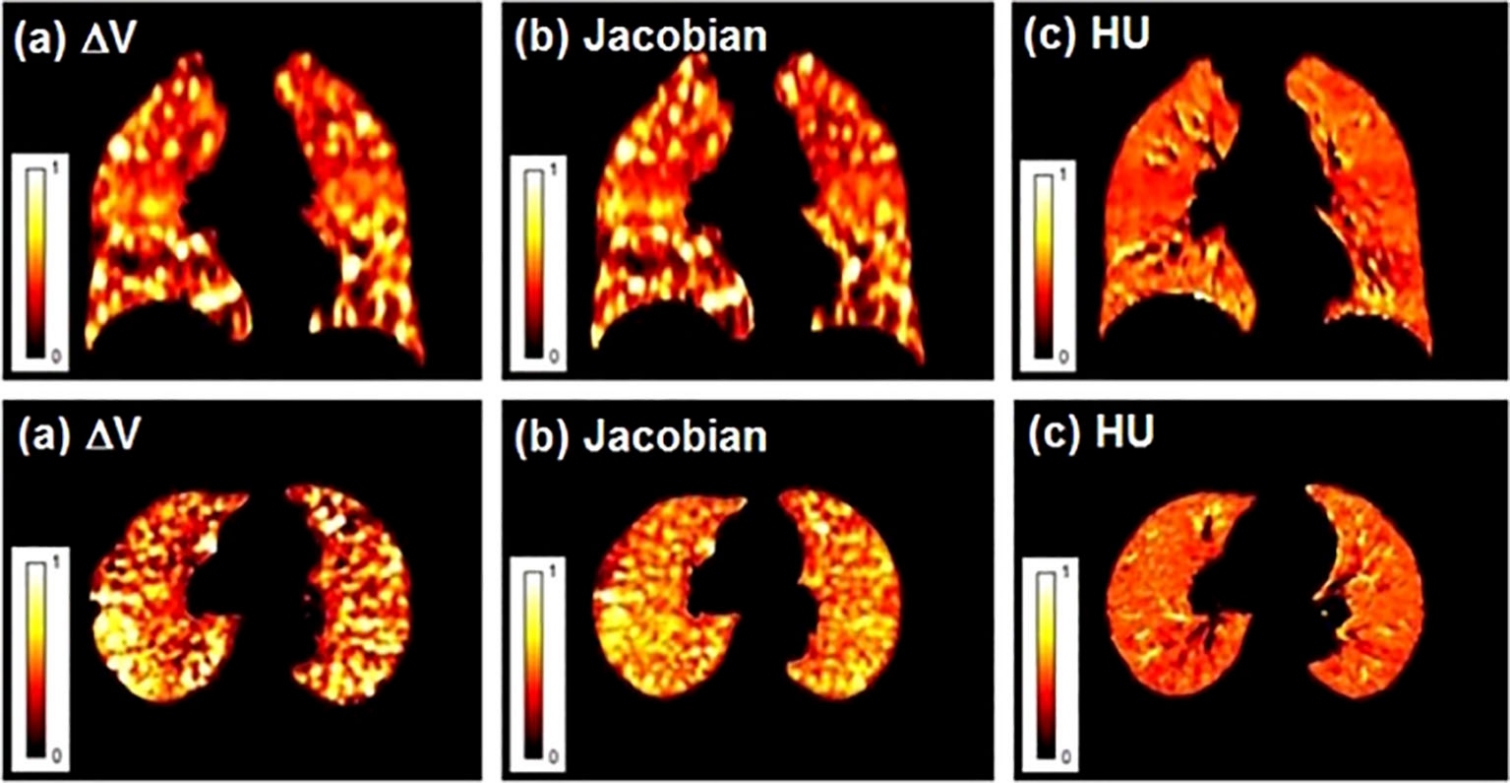
Lung ventilation function images obtained by different ventilation imaging algorithms (VIAs). **(A)** ΔV; **(B)** Jacobian; **(C)** HU. Reproduced from reference (43) with permission from Wiley, copyright 2013.

4DCT lung ventilation imaging involves two main steps, one of which is the quantitative calculation of CT value (HU), Jacobian, or volume of the voxel (ΔV) changes in different respiratory phases (3, 43, 51). Latifi et al. (43) compared the above three VIAs, and the results showed that the similarity between ΔV and Jacobian is higher than that between HU and Jacobian, and ΔV and HU (**Figure 1**). Kida et al. (52) compared the calculation of lung ventilation based on HU and Jacobian with SPECT ventilation, respectively, and confirmed that there are smaller differences and a stronger correlation between HU and SPECT lung ventilation. Recently, Tian et al. (53) proposed a simplified algorithm for calculating lung ventilation (VIA AVG), with higher accuracy, efficiency, and fewer input requirements. Moreover, Liu et al. (54) developed deep learning (DL) method to calculate 4DCT lung ventilation. The results confirmed that compared with the method based on HU and Jacobian, the DL method has the highest similarity with SPECT lung ventilation.

The second is the DIR algorithm, which mainly has two methods: surface-based registration and nonparametric registration based on volume (3). DIR algorithm is one of the cornerstones of 4DCT ventilation, and its accuracy will affect the final ventilation image. Latifi et al. (43) found that HU-based ventilation imaging is less dependent on different DIR algorithms than ΔV-based and Jacobian-based. Kipritidis et al. (55) proposed a simplified HU-based VIA that uses the 4D time-averaged product of regional air and tissue density for each voxel without relying on DIR. Results show that the new HU-based method is more related to the voxel of PET ventilation imaging than the HU-based DIR algorithm. Recently, Zhong et al. (56) developed a method based on deep convolutional neural networks, using inhalation peak and expiration peak phases as input data and HU-based DIR ventilation images as label data for training. The results demonstrate that the predicted ventilation images can be directly derived from 4DCT without explicit image registration and show a high degree of similarity with label data.

### Magnetic resonance imaging

MRI has more advantages than CT, including excellent soft-tissue resolution, no ionizing radiation, the potential to obtain different nuclear information, and provides higher spatial resolution than nuclear medicine imaging (2, 13). Wielputz et al. (57) stated that the performance of MRI to evaluate perfusion, ventilation, and respiratory mechanics is better than MDCT. The main disadvantages of MRI are organ movement, lack of protons in the lung parenchyma, and field inhomogeneities, which significantly reduce the signal-to-noise ratio (11, 58). Several methods have been adopted to overcome these drawbacks in the past few years. A study has shown that Fourier decomposition/matrix pencil MRI (FD/MP-MRI) or hyperpolarized gas can be used to assess lung ventilation, and dynamic contrast-enhanced MRI (DCE-MRI) or no-contrast method (FD/MP-MRI) can be used to assess lung perfusion (59).

**Figure 2:**
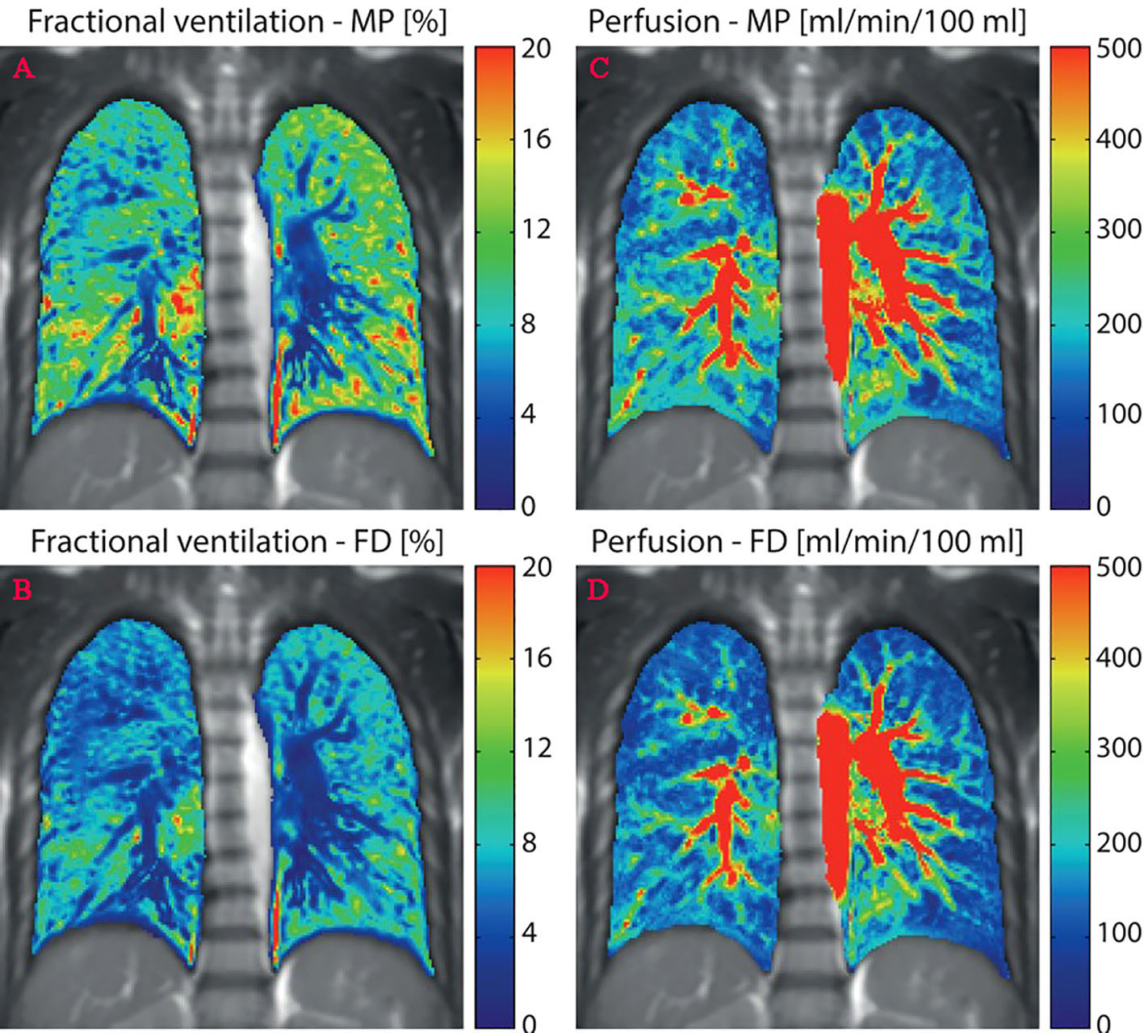
Quantitative maps of ventilation and perfusion calculated using the matrix pencil (MP) and Fourier decomposition (FD) methods. **(A, C)**, MP-MRI ventilation/perfusion; **(B, D)**, FD-MRI ventilation/perfusion. Reproduced from reference (64) with permission from Wiley, copyright 2016.

FD-MRI uses the short-echo method available on the scanner and the weak contrast generated when air enters and leaves the lungs during free breathing to generate lung ventilation maps. Acquire time-resolved two-dimensional datasets to generate perfusion maps without inhalation or injecting the tracer (12, 21). Kaireit et al. (60) confirmed that FD-MRI ventilation imaging and PFT have a good correlation and short-term repeatability (61). Furthermore, Bauman et al. (62) proved the qualitative consistency of FD-MR imaging and SPECT/CT in animal experiments assessing regional lung ventilation and blood perfusion. Other studies have shown that image quality can be improved by using nonuniform FD to replace standard fast FD (63) or ultra-fast steady-state free precession (64). MP-MRI is a derivative of FD-MRI that does not require tracers and respiratory restriction (59, 65). Compared with FD technology, MP decomposition can automatically, reliably, and accurately estimate the modulation amplitude of respiratory and cardiac signals (**Figure 2**) (66). From here, we can know that through FD-MRI or MP-MRI, ventilation and perfusion images can be obtained from one scan without a tracer.

Some inert gases can be artificially hyperpolarized, and a magnetic resonance phenomenon occurs, so it can be used as an inhalable tracer to show lung ventilation (e.g., ^3^He, ^129^Xe, ^19^F) (12, 13, 21). The disadvantage of ^3^He and ^129^Xe is that the magnetization decays quickly and the storage time is short, while ^19^F has sufficient polarization ability for imaging at room temperature, but the image quality is obviously poor, and the inspection time is long at the same resolution (12, 21). Moreover, oxygen itself has paramagnetic properties and can also be directly used for lung ventilation imaging by inhaling 100% oxygen to obtain an image enhancement signal and subtracting the image signal of inhaling room air (20% oxygen) to detect ventilation (21, 58). Doganay et al. (67) stated that hyperpolarized ^129^Xe MRI ventilation is closely related to SPECT/CT ventilation imaging. However, Rankine et al. (68) stated that there are differences in f-MLD and volume of functional lung receiving ≥20 Gy (fV_20_) between the ventilation function represented by ^129^Xe MRI and the functional planning representing the actual regional lung function (gas exchange imaging), and ^129^Xe-MRI-based ventilation function is not an effective substitute for true regional lung function in all patients. All gas-based MRI ventilation imaging has its inherent shortcomings, and it is relatively rare to detect ventilation clinically.

DCE-MRI is an intravenous injection of a paramagnetic contrast agent (e.g., gadolinium-diethylene triamine penta-acetic acid (Ga-DTPA)), resulting in an increase in the signal in the T1-weighted image, which allows the visualization of local perfusion (12, 13). Johns et al. (69) showed that DCE-MRI perfusion has higher sensitivity in detecting chronic thromboembolism pulmonary hypertension compared with SPECT perfusion imaging. In fact, DCE-MRI and FD-MRI have higher consistency in the semiquantitative assessment of lung perfusion, but DCE-MRI has better image quality (70). However, Maxien et al. (71) developed a new DCE-MRI acquisition technology that shows that the free-breathing state does not increase the movement compared with the breath-hold state, and it is more suitable for quantitative assessment of lung perfusion.

### Nuclear medicine imaging

#### Single-photon emission CT

In SPECT, one or more cameras rotate around the patient to generate planar images from multiple angles, which are then used to generate a set of tomography images of the distribution of radiotracers in the lungs (72). In the past 10 years, the emergence of SPECT/CT scanners has not only obtained SPECT ventilation and perfusion imaging data but also obtained CT imaging data of low-dose radiation dose levels for attenuation correction, and it has higher image quality and diagnostic accuracy compared with planar scintigraphy (72, 73). Since radionuclide imaging has been widely used to assess lung function for a long time and maintains relative evaluation standards, SPECT/CT can provide better spatial resolution and 3D anatomical information. So, it has been selected as a reference for assessing lung ventilation and perfusion standards (40).

The radioactive tracers used in SPECT/CT ventilation imaging include gas type (^133^Xe,^81m^Kr), aerosol type (^99m^Tc-DTPA), and solid type (^99m^Tc-Technegas), and the radioactive perfusion tracer uses technetium-99m-labelled macroaggregated albumin (^99m^Tc-MAA) (2, 72–74). However, due to the high cost of radioactive tracer gas, short half-life, and difficulty in storage and collection, the ^99m^Tc-Technegas and ^99m^Tc-MAA are now the most commonly used radiotracers for ventilation and perfusion imaging, respectively (73).

#### Positron emission tomography

Compared with SPECT, both are advanced technologies for image acquisition, but PET has higher sensitivity for radioactive decay detection, higher temporal and spatial resolution, excellent quantification capabilities, and shorter acquisition time (73, 75, 76). It can also be combined with CT scans to obtain higher image resolution and is currently commonly used in lung cancer radiation therapy planning (77). Even respiration gating can be used to reduce respiratory motion artifacts, and 4D ventilation and 4D perfusion of PET/CT can even be obtained by combining 4DCT (11, 73, 75). In the past, most PET radiotracers used to study lung physiology only had a short lifetime (such as ^15^O, ^13^N, and ^11^C), but now they are replaced by Galligas and Gallium-68 macroaggregated albumin (^68^G-MAA) for lung ventilation and perfusion, respectively (2, 75, 76, 78). The study by Le Roux et al. (79) found a strong correlation between PET/CT pulmonary function and PFT parameters, and they then proposed an automatic segmentation method that can be used for semiautomatic delineation (80, 81).

## Clinical practice

### Reducing functional lung radiation dose

Multiple functional lung imaging modalities have been applied to the clinic to optimize radiotherapy planning and reduce the radiation dose of functional lungs. Yamamoto et al. (82) compared the difference between the 4DCT ventilation function image-guided IMRT planning (f-IMRT) and the anatomical planning for patients with stage IIIB nonsmall cell lung cancer (NSCLC). The results showed that lung fV_20_ decreased by 5% while maintaining the target dose and meeting the dose limits of other critical organs. Wang et al. (83) showed that the five manually optimized beams for functional lung protection IMRT plans were more effective in reducing the dose to functional lungs compared to the five equally spaced beams for functional lung protection IMRT plans. As for integrating MRI-based functional lungs into clinical radiotherapy planning, a randomized, double-blind trial of functional lung avoidance based on ^3^He ventilation to evaluate pulmonary toxicity was prepared to achieve a reduction of 1.5 Gy and 3% in f-MLD and fV_20_, respectively (84). A meta-analysis based on utilized functional lung imaging modalities (including CT, 4DCT, SPECT, and PET) showed that f-MLD and fV_20_ decreased by 2.2 Gy and 4.2% when optimizing functional lung protection planning compared with conventional anatomical CT planning (more details in reference 17; Table 3) (17).

Bates et al. (85) stated that compared with the anatomical IMRT planning, the fV_20_ and f-MLD of the f-IMRT planning (based on SPECT/CT) were reduced by 2.5% and 0.4 Gy, respectively. Combining PET/CT ventilation/perfusion into radiotherapy planning, Siva et al. (86) studied the 30% maximum standard uptake value (SUV max) of 4D-PET/CT perfusion to determine the well-perfused lung volume. They optimized the 3D-CRT to protect the well-perfused lung volume. As a result, the functional V_30_, V_40_, V_50_, and V_60_ were improved, and the f-MLD of the lung improved by 0.86 Gy. In another study, the author found that the 70% SUV max threshold of the 4D-PET/CT scan was used to describe the “highly perfused” (HP-Lung) and “highly ventilated” (HV-Lung) lung volumes and used this to optimize IMRT planning. The results showed that HP-Lung-based f-MLD was significantly reduced by 13.0% (1.7 Gy), and fV_5_, fV_10_, and fV_20_ were improved by 13.2%, 7.3%, and 3.8%, respectively. At the same time, the LDVP in HV-Lung was not significantly different from the anatomical planning (87). A meta-analysis based only on PET/CT and SPECT/CT indicated that the integration of perfusion images into the radiotherapy planning of lung cancer patients could improve functional LDVP compared to conventional anatomical CT planning (f-MLD and fV_20_ decreased by 0.24 Gy and 0.41%, respectively), but ventilation also did not show significant improvement (88).

**Figure 3:**
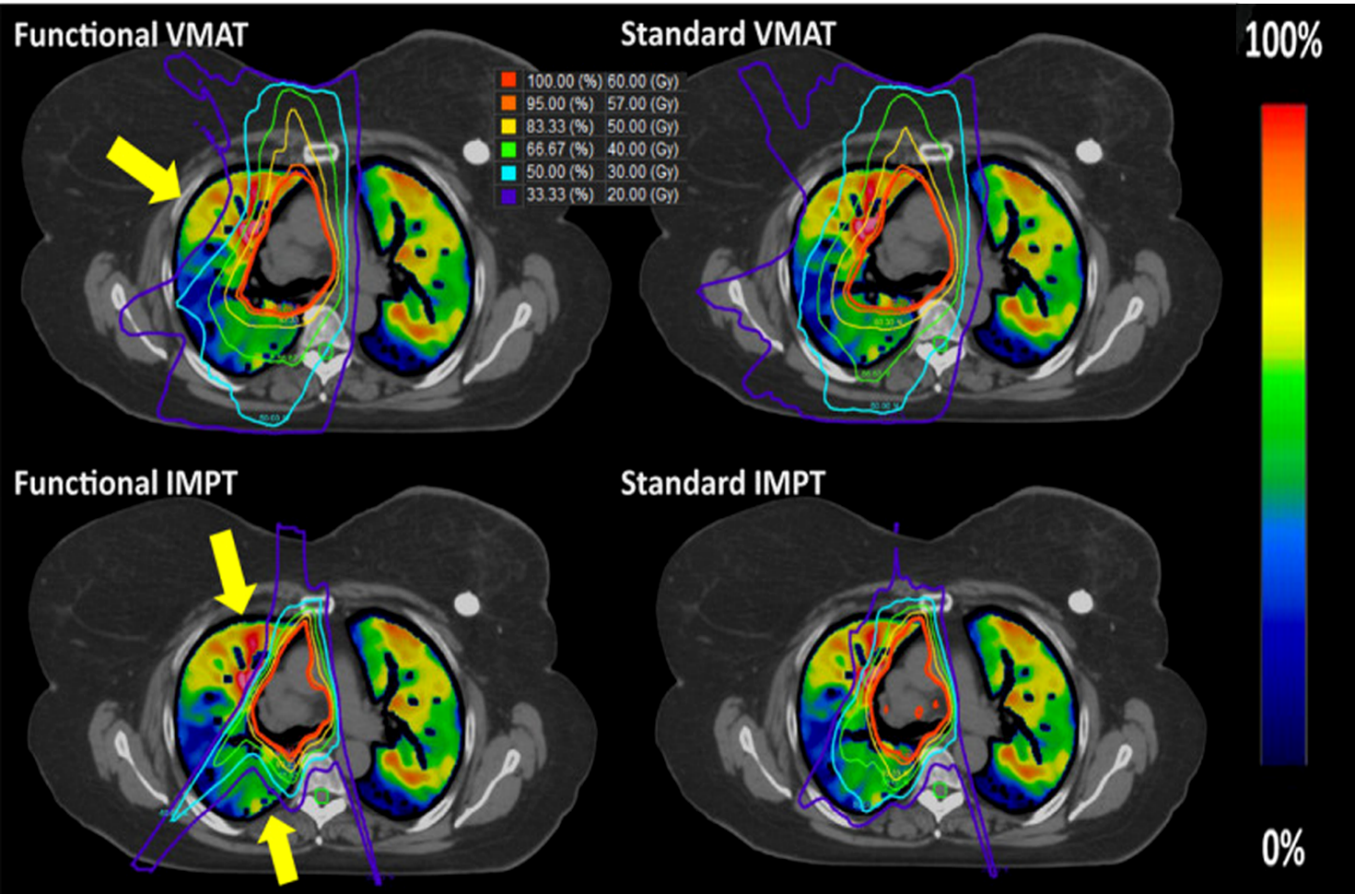
Examples of the standard and functional plans with VMAT (top) and three-field IMPT (bottom) techniques. The yellow arrow indicates where the most functional sparing occurs for this patient. Reproduced from reference (90) with permission from Wiley, copyright 2021.

Not only that, but functional lung imaging has also been extended to proton therapy (PT). Leko et al. (89) compared the dosimetric differences between three-dimensional conformal radiotherapy (3D-CRT), VMAT, and PT functional lung protection plans guided by 4DCT ventilation images. The results showed that PT compared with VMAT and 3D-CRT, the fV_5_ was 10.7%, 21.9%, and 26.0%, and the f-MLD was 3.8, 5.2, and 5.6 Gy, respectively, which indicate that the PT-based functional lung sparing planning can further reduce the functional lung dose (example shown in **Figure 3**). The functional IMPT (intensity-modulated proton therapy) plans show significant dose reduction when compared to the functional VMAT plans for the f-MLD at 14.49 Gy vs. 7.31 Gy, fV20 at 22.66% vs. 13.48% (90) Huang et al. (44) further compared two functional proton plans based on 4DCT ventilation images (double scattering proton therapy (DSPT) and intensity-modulated proton therapy (IMPT)) with IMRT in preserving high-functioning lungs. The results show that DSPT and IMPT plans are better than IMRT in protecting the low-dose areas of the total lung (V_5_), and functional DSPT and functional IMPT have benefits in protecting high-functioning lungs, respectively, compared with their anatomical planning.

### Predicting radiation pneumonitis

In the past, lung dose-volume parameters (LDVP) such as MLD, V_5_, and V_20_ were used to predict the occurrence of radiation pneumonitis, but since the introduction of functional planning, the occurrence of radiation pneumonitis can be predicted more accurately through functional LDVP (f-MLD, fV_20_, etc.). Farr et al. (91) study showed that the area under the curve (AUC) for f-MLD and MLD in predicting G2+ radiation pneumonitis was 0.812 and 0.716, respectively (the same for fV_20_ and V_20_ was 0.792 and 0.716, respectively). Similar results were obtained in another study (92). Recent studies have shown that SPECT/CT-based ventilation and perfusion functional LDVP are used to predict RILI, indicating that if the patient has central-type NSCLC or chronic obstructive pulmonary disease, ventilation-based (V-fV_20_, V-f-MLD) is better than perfusion-based (Q-fV_20_, Q-f-MLD) to predict RILI (93).

Although it is generally said that functional LDVP can more accurately predict RILI, it is not clear what the threshold for defining functional lung can be a more accurate prediction. Ding et al. (94) seem to answer this question; that is, when 20% of the maximum value of SPECT/CT lung perfusion image count was used as the threshold, the highest predictive ability was obtained with fV_20_ of 0.928. However, it is true that there is no consensus on the optimal threshold for other functional imaging modalities.

### Reducing radiation pneumonitis incidence

Regarding the risk of pulmonary radiation toxicity, Faught et al. (95) found that using 4DCT lung ventilation function imaging-guided IMRT planning can decrease 2+ and 3+ grade radiation pneumonitis by 7.1% and 4.7% (based on Normal tissue complication probability models), respectively.

According to the recent phase II clinical trial, Vinogradskiy et al. (96) showed that the 4DCT ventilation function image-based functional lung protection planning reduced the incidence of G2+ radiation pneumonitis to 14.9% in lung cancer patients receiving curative chemoradiotherapy (lower than the historically reported 25%), which did meet the expected phase II criteria (NCT02528942). Currently, clinical trials that have been registered at ClinicalTrials.gov for functional lung protection planning-guided radiotherapy are summarized in **Table 1**.

**Table 1:** Registered clinical trials of functional lung sparing planning-guided radiotherapy (from ClinicalTrials.gov).

Author	ID	Phase	Statue	Last updated	Characteristics	Modalities	Objective (lung)	Institutions
Huang et al. (97)	NCT05134558	–	Recruiting	November 2021	Lung cancer 60 (20–80 years) RT (–)	CT (Xenon)	RILI	National Taiwan University Hospital
Filion et al. (98)	NCT04863027	I–II	Recruiting	April 2021	Lung cancer 60 (≥18 years) RT or SBRT (–)	DECT	RILI	Centre Hospitalier de l’Université de Montréal
Yamamoto et al. (99)	NCT02308709	–	Active, not recruiting	August 2021	Lung cancer 33 (18–80 years) RT or SABR (–)	4DCT	RILI PFT, PDFT	University of California, Davis
Hsu et al. (100)	NCT03077854	–	Recruiting	March 2019	NSCLC (III) or SCLC (Limited Stage) 64 (>20 years) RT (54–60 or 45 Gy)	4DCT	RILI PFT, PDFT	National Taiwan University Hospital
Bayouth et al. (101)	NCT02843568	–	Recruiting	June 2021	NSCLC 139 (>18 years) RT (60–66G y) or SBRT (40–60 Gy)	4DCT	RILI PFT, PDFT	University of Wisconsin Carbone Cancer Center
Vinogradskiy et al. (96, 102)	NCT02528942	II	Completed	July 2021	Lung cancer 101 (>18 years) RT (45–75 Gy)	4DCT	RP (2+)	University of Colorado Cancer Center
Yaremko et al. (84, 103)	NCT02002052	II	Terminated (insufficient)	September 2019	NSCLC (III A-B) 29 (>18 years) RT (60 Gy)	MRI (^3^He)	RILI	London Health Sciences Centre
Zeng et al. (104)	NCT02773238	II	Active, not recruiting	January 2022	NSCLC (II B–III B) 56 (≥18 years) RT (60 Gy)	SPECT/CT (^99m^TC-SC, ^99m^TC-MAA)	RP (2+) PFT, PDFT	University of Washington
Farr et al. (105)	NCT01745484	–	Completed	April 2019	NSCLC (I–III) 71 (>18 years) RT (–)	SPECT/CT (–)	RP (2+)	Aarhus University Hospital
Farr et al. (106)	NCT04676828	II	Recruiting	January 2021	Lung cancer 195 (>18 years) RT (60–66 Gy)	SPECT/CT (–)	RP (2+)	Aarhus University Hospital
Rachel et al. (107)	NCT04942275	II–III	Recruiting	July 2021	NSCLC 60 (>18 years) SBRT (–)	PET/CT (^68^Ga-MAA)	RILI PFT	University Hospital, Brest
Bucknell et al. (108)	NCT03569072	–	Recruiting	September 2020	NSCLC (III A–C) 20 (>18 years) RT (69 Gy)	PET/CT (^18^F-FDG)	RILI	Peter MacCallum Cancer Centre

NSCLC, nonsmall cell lung cancer; RT, radiotherapy; SBRT, stereotactic body radiotherapy; SABR, hypofractionated stereotactic ablative radiotherapy; PFT, pulmonary function test; PDFT, pulmonary diffusion function test; RILI, radiation-induced lung injury; RP, radiation pneumonitis; ^99m^TC-SC, technetium Tc-99m sulfur colloid; ^99m^TC-MAA, technetium Tc-99m albumin aggregated; ^68^Ga-MAA, human albumin macroaggregates labeled with Ga-68; ^18^F-FDG, 18F-2-fluoro-2-deoxy-d-glucose fluorodeoxyglucose.

## Current problems and future research directions

**Figure 4:**
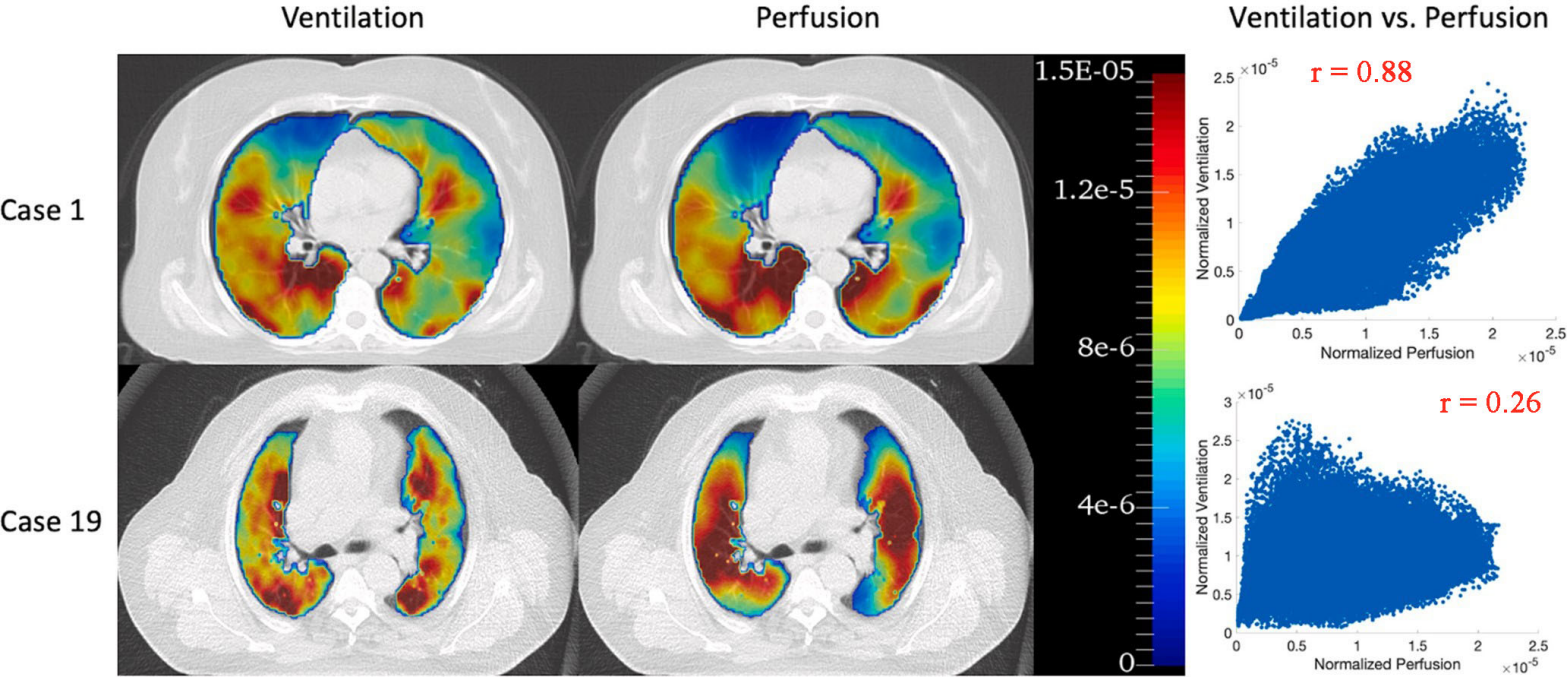
^68^Ga PET/CT ventilation and perfusion images and voxel-wise correlations between ventilation and perfusion for two representative patients: case 1 with the highest correlation (*r* = 0.88) and case 19 with the lowest correlation (*r* = 0.26). Reproduced from reference (111) with permission from Elsevier, copyright 2022.

1. Which functional lung imaging modalities and parameters should be selected? Although CT imaging is the gold standard for radiotherapy plan design and can save time and cost, MRI does not involve ionizing radiation and improves soft tissue contrast (109), and the reference standard for functional lung imaging is SPECT/CT and PET/CT (40).
2. Choose functional lung imaging-guided radiotherapy plan optimization based on ventilation or perfusion or ventilation-perfusion ratio. There have been many studies showing that no matter whether ventilation-based or perfusion-based imaging is included in the functional lung protection planning-guided radiotherapy, it is beneficial to reduce the functional lung dose, while Yuan et al. (16) study showed that only 61% of patients included in the study have ventilation and perfusion defects matching, the application of SPECT/CT ventilation-perfusion ratio to protect the functional lung will change the functional lung protection plan based on perfusion alone in 39% of patients. Forghani et al. (110) also reported that approximately 25% of patients with stage III lung cancer showed lower agreement in SPECT/CT ventilation and perfusion (example shown in **Figure 4**).

As mentioned above, ventilation and perfusion need to be maintained in an appropriate ratio to facilitate gas exchange (represent real lung function). Therefore, is it necessary to acquire both lung ventilation and perfusion images when designing functional lung protection radiotherapy planning.
3. Is it necessary to divide the functional lung into different areas according to different thresholds to optimize radiotherapy planning? Yuan et al. (16) and Meng et al. (112) divided the total lung function into five areas according to different underlying causes and thresholds (type A: tumor occupied lung regions; type B1: complete function defect areas induced by unrecoverable diseases; type B2: reduced lung function areas induced by unrecoverable diseases; type B3: temporarily dysfunctional lung due to tumor or other potentially reversible conditions; type C: normal functional lung). However, previous studies usually directly divide the whole lung into two areas: functional lung and non-functional lung (113). Moreover, Shioyama et al. (114) used 50% and 90% of the maximum count of SPECT/CT perfusion images as thresholds for f-IMRT planning (f-IMRT_50%_, f-IMRT_90%_), and compared their differences with anatomical planning. The results showed that f-MLD and fV_20_ were reduced by 2.2 and 4.2 Gy and 5.1% and 6.8%, respectively (f-IMRT_50%_ vs. f-IMRT_90%_). So, whether the planning is designed to protect one threshold-defined functional lung or to divide the whole lung into multiple regions with different functional states for separate protection (multiple threshold-defined), the planning must be carefully considered.
4. When radiotherapy restores temporary lung function defects caused by tumors or other potentially reversible diseases, is it necessary to redesign the functional lung protection radiotherapy planning? What is then the best time to re-planning? Yuan et al. (115) observed that lung cancer patients’ local ventilation and perfusion were significantly improved when the radiotherapy reached 45 Gy compared to before the radiotherapy. In addition, Meng et al. (112) stated that potential changes in these functional lung regions could affect lung dose. Moreover, Yamamoto et al. (116) used two re-planning to protect the functional lung (two-time points are 16–20 Gy and 30–34 Gy), and the cumulative dose of the functional lung (f-MLD) was further reduced than that without re-planning (reduced by 5.0% and 3.6%, respectively). This shows that re-planning is necessary, but there is no uniform standard for the timing and frequency of re-planning.

## Conclusion

The research of lung ventilation or perfusion imaging has been applied to clinical diagnosis for a long time. The combination of lung function imaging into radiotherapy planning has also attracted widespread attention, and studies continue to report new modalities and clinical application values of functional lung imaging (e.g., predict RILI, decrease the incidence of RILI). For DECT and 4DCT, it may be a better conventional method for pulmonary function imaging because the radiotherapy planning is based on CT as the gold standard. Imaging can be performed during disease diagnosis or simulated positioning without too much time and cost. DECT can obtain ventilation and perfusion images simultaneously by inhaling krypton and an intravenous injection of an iodine-containing contrast agent. However, the functional lung obtained by the above imaging modalities has not been physiologically verified, and the optimal imaging modalities and functional lung definition thresholds have not been clearly determined. We need to perform further verification tests and clinical trials (lack of phase III clinical trial results), but it is undeniable that the existing research evidence shows that the planning of integrated functional lung imaging to protect the functional lung is beneficial to reduce the occurrence of radiation-induced lung injury.

## Author contributions

All authors listed have made a substantial, direct, and intellectual contribution to the work, and approved it for publication.

## Conflict of interest

The authors declare that the research was conducted in the absence of any commercial or financial relationships that could be construed as a potential conflict of interest.

## Publisher’s note

All claims expressed in this article are solely those of the authors and do not necessarily represent those of their affiliated organizations, or those of the publisher, the editors and the reviewers. Any product that may be evaluated in this article, or claim that may be made by its manufacturer, is not guaranteed or endorsed by the publisher.
